# Worldwide survey of T2* cardiovascular magnetic resonance in Thalassaemia

**DOI:** 10.1186/1532-429X-13-S1-O15

**Published:** 2011-02-02

**Authors:** John-Paul Carpenter, Taigang He, Paul Kirk, Lisa Anderson, John B Porter, Malcolm Walker, Renzo Galanello, Fabrice Danjou, Gianluca Forni, Antonis Kattamis, Vassilis Ladis, Marouso Drossou, Demetra Vini, Andreas Michos, Vassilios Perifanis, Tuncay Hazirolan, Ana Almeida, Yesim Aydinok, Mirella Rangelova, Amal El-Beshlawy, Mohsen Elalfy, Ibrahim Alnasser, Shahina Daar, Juliano de Lara Fernandes, Dudley J Pennell

**Affiliations:** 1 Royal Brompton and Harefield NHS Trust, London, UK; 2 St. George's Hospital NHS Trust, London, UK; 3 University College Hospitals NHS Trust, London, UK; 4 Ospedale Regionale Microcitemie, Cagliari, Italy; 5 Ospedali Galleria di Genova, Genova, Italy; 6 Aghia Sophia Children's Hospital, Athens, Greece; 7 General Hospital of Piraeus, Athens, Greece; 8 General Hospital of Corfu, Corfu, Greece; 9 Hippokration General Hospital, Thessaloniki, Greece; 10 Hacettepe Medical University, Ankara, Turkey; 11 University Hospital Santa Maria, Lisbon, Portugal; 12 Ege Univeristy Medical Faculty, Izmir, Turkey; 13 National Centre of Haematology, Sofia, Bulgaria; 14 Cairo University Hospital, Cairo, Egypt; 15 Ain Shams University Hospital, Cairo, Egypt; 16 King Fahad Armed Forces Hospital, Jeddah, Saudi Arabia; 17 Sultan Qaboos University, Musat, Oman; 18 University of Campinas, Sao Paolo, Brazil

## Introduction

Thalassaemia major (TM) affects hundreds of thousands of patients worldwide but only a minority have access to regular blood transfusion and chelation therapy. Cardiovascular magnetic resonance (CMR) T2* measurement provides an accurate, reproducible measurement of cardiac iron which is the cause of heart failure and early death in many transfused TM patients. This technique has been adopted as part of routine management in many countries where survival is now approaching normal but little is known about the severity and effects of myocardial iron loading in different geographical regions.

## Purpose

The aim of this study was to describe the burden of disease of myocardial siderosis (measured by T2*) in different populations throughout the world and to assess the relationship between T2* and outcome such as heart failure and cardiac death.

## Methods

34 worldwide centres were involved in this survey of 3376 patients from Europe, the Middle East, North America, South America, North Africa, Australia and Asia. Anonymised data on myocardial T2* values were analysed in conjunction with clinical outcomes (heart failure and death).

## Results

Overall, 57.5% of patients had no significant iron loading (T2* >20ms), 22.6% had moderate cardiac iron (10ms<T2*≤20ms) and 19.9% had severe cardiac iron (T2* ≤10ms) at baseline. The lowest burden of iron was found in North Africa, North and South America with an intermediate level in Australia, Europe and the Middle East but a high proportion of patients (>50%) in South-East Asia had cardiac iron (T2* >20ms) at baseline. At the time of the first scan, 100 patients (3.3%) had confirmed heart failure, the majority of whom (77.0%) had myocardial T2* <10ms with almost all (99%) having T2* <20ms. There were 113 patients who subsequently developed heart failure. 92.0% of these had T2* <10ms and 99.1% had a T2* <20ms. There were 39 deaths. Cardiac T2* values were <10ms in 79.5%, with 92.3% <20ms.

## Conclusions

Even in this well-treated cohort with access to transfusion, chelation and CMR, there is a large proportion of TM patients with moderate to severe cardiac iron loading. Low T2* (<10ms) is associated with cardiac failure and death. There is a huge unmet worldwide need in terms of access to specialist medical care (including transfusion and chelation therapy) together with advanced monitoring techniques (such as CMR).

